# Pyorrhœa Alveolaris

**Published:** 1890-02

**Authors:** 


ARTICLE II.

### PYORRHŒA ALVEOLARIS.

Dr. James Truman.—Mr. Chairman, I am placed in rather an embarrassing position, in having to talk to an audience composed largely of gentlemen who did not hear Dr. Allan's essay this morning, and many of whom probably have come to listen to the paper that will succeed my remarks; however, I will endeavor to condense as much as possible, what I have to say in answer to Dr. Allan's paper.

The first point that seemed to me to require attention was his statement that the investigations of Dr. Black, of Illinois have given us all that we really know in regard to pyorrhœa alveolaris. Now as this question has occupied the professional mind for the last twenty or twenty-five years, I do not think the statement can be wholly relied upon as true. No doubt, Dr. Allan thinks it is true. I am not willing to count myself second to any one in appreciation of Dr. Black's histological work; but long preceding his investigations, I think, the profession quite thoroughly understood the nature of this pathological condition. Twenty years ago I spent a great deal of time in the investigation of this subject, following the lead of the French workers, especially Dr. Magitot; but the results of treatment were not satisfactory.

Dr. Allan stated this morning that the loss of teeth from pyorrhœa alveolaris is greater, probably than from all other causes combined, if I understood him correctly. That certainly is a mistake. It depends largely upon his definition of pyorrhœa alveolaris. Allowing the largest latitude for that definition, taking in all the senile teeth, and including all those that have suffered absorption through the action of tartar, as well as those that can be justly said to be destroyed by this disease, and we would not have a number that would at all compare, in my judgement, with the number destroyed by caries. But has he any right to take in senile teeth, and the teeth that have been destroyed by tartar? I contend that those teeth that have been injured by deposits, or lost in old age, where absorption has taken place, cannot be included; their loss is due to an entirely different action. Also those teeth that have been lost or loosened by salivation are, in my view, to be thrown out entirely; and we should then include only those that are affected by the pathological condition which we term pyorrhœa alveolaris.

The essayist took the position, if I understood him, that the presence of pus does not represent the disease. Now what is pus? If I understand at all the origin of pus, and perhaps I do not, it is this: irritation precedes what we call inflammation, and inflammation, produces what we term pus, from two processes: first, emigration of the so-called white blood-corpuscles; and, secondly, the breaking down of tissue and the retrograde metamorphosis of the tissue to its original protoplasmic elements. It is possible to have inflammation without one or both of these processes going on? I certainly contend that it is not. The moment you have irritation you have an enlargement of the blood-vessels and a congested condition, and when that occurs, you have the emigration of the white blood-corpuscles, and eventually a breaking down of the tissue and the retrograde metamorphosis that I spoke of. Can we have inflammation, such as we understand by pyorrhœa, without pus? Certainly it does not appear to me to be so.

The essayist further stated that the name was a misnomer. That is, in one sense, true. But some words change their meaning. All words represent an idea, and that idea may change in the course of time. When the French writers named this disease pyorrhœa alveolaris, it meant to them precisely what the term conveyed—pus originating from the alveolus, the bone surrounding the teeth. In the course of time that idea has changed, but the name remains; and I think it is well to continue it. If Dr. Allan had been a teacher as long as I have, probably he would understand how difficult it is to go before a class of young men and talk on subjects, and have to enter into a definition of the different terms, which have been given by various individuals when they had found those that represented their ideas, little thinking that in the course of time these will change, and their terms may no longer represent these pathological conditions. As a matter of fact, is this name entirely wrong? Dr. Black, whose work in this direction we all fully appreciate, has shown us most clearly that the periosteum is made up of inelastic fibers, that extend from the cement on the one hand to the bone on the other without break; and if inflammation occurs in the periosteum, it is necessarily carried to the cement on the one side and to the bone on the other. Therefore is it wrong to call this disease pyorrhœa alveolaris? I do not think it is. Nine times out of ten, the essayist stated, tartar was the cause of the disease. Now, if nine times out of ten tartar is the cause of this disease, we ought to have it in all cases where tartar is found. He acknowledged that ordinary salivary calculus was not the cause of this disease; but the tartar which he found upon the teeth or roots he called serumal tartar. He has taken up the old, old song of serumal tartar, and affirms dogmatically that it is serumal tartar. I have yet to learn of a single investigation that has been made to demonstrate the existence of this characteristic tartar. What is it? If I understand it, it means a calcareous deposit from the serum of the bloob. We all know very well that there is scarcely an organ in the body that may not receive such deposits; but we have no evidence that this serumal tartar, so-called, comes from the serum of the blood. May it not come from pus? because pus can deposit calcareous particles as well as any fluid of the blood. As long as we do not know that it does come from the serum of the blood we have as much right to affirm—as this gentleman has to assume the contrary—that it is derived, as ordinary tarter, from the salva.

Now, what is the genesis or origin of this pathological condition? Does it originate from tartar? Not if I understand it. Does it originate from the roughness that Dr. Allan spoke of, at the gingival border of the teeth? Possibly; but where does that roughness come from? When you take a patient in hand, and that patient states to you that in the morning when he brushes his teeth the blood will ooze from the gum, "that his teeth bleed," you know, and we all know, what that condition is. Here and there a tooth will present a bright red line at the border-line of the gum. The moment that is touched blood will ooze from it, by the disturbance of the capillaries at that point. That is the beginning. And if you take it in hand at that stage you can stop pyorrhœa alveolaris. It has nothing to do with tartar. It may come from constitutional disturbances; it may come from some form of nephritis, or a long siege of sickness. What then follows necessarily after this? Immediately succeeding we have a development of micro-organic life. That I demonstrated twenty years ago. On examination of these cases I found large quantities of bacteria throughout the broken-down tissue. I soon learned that they had something to do, although I did not then appreciate the full extent of their action, with all these inflammatory conditions; and you dentists, every one of you, can and do make those conditions in the mouths of your patients by carless work. When you place a rubber dam, or a clamp, or ligatures upon teeth, you produce irritation, and the patient will complain. You take off the instrument, and you allow the patient to go away without any treatment whatever. In forty-eight hours there will be a development of micro-organisms, and pain will result, and irritation at the neck of the tooth; and if it is not stopped at that time, it may go on until this pathological condition which we call pyorrhœa alveolaris appears.

What is the treatment? First, I hold that no dentist should put a rubber dam in the mouth, or a clamp on the teeth, or do any thing of that kind that is liable to raise inflammation at the necks of the teeth, without applying an antiseptic. For this purpose I know nothing better than sulphate of quinia, mixed into a paste—not because it is the best germicide, but because it is more lasting than other agents.

This disease has its origin in inflammation of the periosteum, or pericementum, of the roots of the teeth. There is no question about that. I am satisfied, by the investigations of others, that I have always been right in that respect, and I have been teaching it for many years. It naturally follows, therefore, that if we are to treat the teeth properly we must direct our attention to the micro-organic life first, and not to the tartar, which is secondary. Has Dr Allan ever found his so-called tartar below the line of healthy pericementum? I cannot say that I ever did. The tartar comes in afterwards; first, disturbance of the pericementum then the deposit of tartar. Remove that tartar and what is the result? Always a necrosed tissue. I have never succeeded in building up the periosteum beyond that line. Dr. Atkinson asserts that he can build the periosteum up to the gingival border of a tooth. I never have been able to do it. If there is tartar there, it should be removed, but it is not the original cause of the trouble; and Dr. Allan himself says that the roots of teeth that have been lost through this pathological condition are shiny and without periosteum. If that is the case, how is he going to do anything with it? The use of mechanical instruments should be secondary. I have treated successfully many cases of pyorrhœa alveolaris, and rarely have I used an instrument. I do not find so much of that tartar as some seem to do. I find it on senile teeth; but that is not pyorrhœa. I do not think any man here ever saw a case of pyorrhœa alveolaris on the lingual surface of the inferior central incisors. You find it on the anterior surfaces but not on the posterior; because the tartar is there to protect the lingual surface. Where tartar is, there cannot arise—does not arise—the pathological condition, in my judgement.

The treatment is necessarily very much in accordance with that stated by Dr. Allan. I do not differ with him materially upon the subject generally. The points of variance I have endeavored to bring before you. In the treatment, it is necessary, first to remove any foreign body that may be present, whether food or other deposits; then inject into the pocket peroxide of hydrogen. I follow that up with sulphuric acid. I was very glad to find that Dr. Allan had been using altogether the commercial sulphuric acid. I long ago abondoned the use of aromatic sulphuric acid, as not being adapted to our purpose. I think we owe the introduction of this agent to Dr. Atkinson. I have used it for many years in this particular work. Magitot recommended chromic acid. I never had any satisfaction in its use, nor any of those more powerful agents. I use the ordinary commercial sulphuric acid, but stronger than Dr. Allan uses it; a twenty-five per-cent solution, and sometimes even stronger than that; but I do not allow it to remain on long. I apply it with a sharp stick around the teeth. It, of course, turns the parts of a dark color. I allow it to remain two or three minutes, not more, just sufficient to burn out the dead tissue; then immediately apply bicarbonate of soda, which brings away every portion of the dead matter. After a little time has elapsed I wash it out with warm water; then apply sulphate of quinia. I have used this for years successfully. If you want to know the philosophy of it, look into the materia medica books, and you will find it described. The sulphate of quinia will remain there longer than any other antiseptic that I know of. If the pain returns in the course of a few days, I would, of course, repeat the sulphuric acid treatment, and after that continue the antiseptic treatment. Then, after the parts have become perfectly healthy, you must use an antiseptic wash to keep them in proper condition. If the pocket remains, there will be a return of the disease, in spite of all your efforts, if you do not take necessary precautions to obliterate it.

This mode of treatment is not original with me, except the bicarbonate of soda and quinia. I have used these for several years with the most decided success and the greatest satisfaction.—*International Dental Journal.*

